# Correction and Removal of Expression of Concern: Highly stereoselective gram scale synthesis of all the four diastereoisomers of Boc-protected 4-methylproline carboxylates

**DOI:** 10.1039/d4ra90023e

**Published:** 2024-03-18

**Authors:** Kehuan Sun, Cheng Tao, Bohua Long, Xiaobin Zeng, Zhengzhi Wu, Ronghua Zhang

**Affiliations:** a College of Traditional Chinese Medicine, Jinan University Guangzhou 510632 China; b Integrated Chinese and Western Medicine Postdoctoral Research Station, Jinan University Guangzhou 510632 China; c The First Affiliated Hospital of Shenzhen University Shenzhen 518035 China bhlong121@163.com; d Shenzhen Institute of Geriatric Medicine Shenzhen 518035 China; e Central Center Lab of Longhua Branch, Department of Infectious Disease, Shenzhen People's Hospital, 2nd Clinical Medical College of Jinan University Shenzhen 518120 Guangdong Province China

## Abstract

Correction and Removal of Expression of Concern for ‘Highly stereoselective gram scale synthesis of all the four diastereoisomers of Boc-protected 4-methylproline carboxylates’ by Kehuan Sun *et al.*, *RSC Adv.*, 2019, **9**, 32017–32020, DOI: https://doi.org/10.1039/C9RA06827A.

The authors regret that in the original ESI several impurity peaks were removed from the NMR spectra.

The authors have repeated their results and a new ESI file with new NMR spectra has been provided.

An independent expert has viewed the original and new spectra and has concluded that they are consistent with the discussions and conclusions presented.

This correction supersedes the information provided in the Expression of Concern related to this article.

The Royal Society of Chemistry apologises for these errors and any consequent inconvenience to authors and readers.

## Supplementary Material

